# Triple therapy for Helicobacter pylori eradication and the risk of hypoglycemia in patients with diabetes: a population-based cohort study

**DOI:** 10.1186/s12889-023-16689-2

**Published:** 2023-09-12

**Authors:** Anne Chang, Anna Chang, Wan-Ting Chen, Lung Chan, Chien-Tai Hong, Li-Nien Chien

**Affiliations:** 1 Department of Endocrinology, Shuang Ho Hospital, Taipei Medical University, New Taipei City, Taiwan; 2 Department of Neurology, Shin Kong Wu Huo-Shih Memorial Hospital, Taipei, Taiwan; 3 Health Data Analytics and Statistics Center, Taipei Medical University, Taipei, Taiwan; 4 Department of Neurology, Taipei Medical University-Shuang Ho Hospital, No. 291, Zhongzheng Rd, Zhonghe District, New Taipei City, 23561 Taiwan; 5 Department of Neurology, School of Medicine, College of Medicine, Taipei Medical University, Taipei, Taiwan; 6 Institution of Health and Welfare Policy, College of Medicine, National Yang Ming Chiao Tung University, Taipei, Taiwan; 7 Graduate Institute of Data Science, College of Management, Taipei Medical University, Taipei, Taiwan

**Keywords:** Hypoglycemia, H.pylori eradication, Type 2 diabetes mellitus

## Abstract

The incidence of type 2 diabetes mellitus has risen globally, from 108 million cases in 1980 to 422 million cases in 2014. Although controlling glycemic levels in patients with diabetes is crucial, insulin and sulfonylureas can cause hypoglycemic episodes and even potentially fatal events such as comas, seizures, life-threatening arrhythmias, and myocardial infarctions. Several antibiotics have been documented to cause hypoglycemic episodes; the use of antibiotics along with insulin or sulfonylureas might further increase the risk of hypoglycemia. Therefore, researchers must determine which antibiotics carry a risk of inducing severe hypoglycemic events. The prevalence of *H. pylori* infection remains high in most countries, and the infection is often treated with triple therapy involving amoxicillin, clarithromycin, and a proton pump inhibitor (PPI). Several case reports have reported that hypoglycemia can occur when used with patients who also take diabetes medication. Therefore, we hypothesized that patients with diabetes have an increased risk of hypoglycemic episodes when being treated with triple therapy for *H. pylori* infection. By analyzing medical records from Taiwan’s National Health Insurance Research Database, we found a significant association between hypoglycemia and triple therapy treatment for diabetic patients with peptic ulcer disease. Prescribing triple therapy to patients with diabetes and peptic ulcers significantly increased the risk of a hypoglycemic episode (adjusted odds ratio [aOR] = 1.75, 95% confidence interval [CI]: 1.64 to 1.88, *P* < 0.001). Similarly, the highest aOR (5.77, 95% CI 4.82 to 6.92) was found in patients with diabetes and peptic ulcers who had hypoglycemic episodes within 30 days after triple therapy treatment. Many patients with diabetes require *H.pylori* eradication for peptic ulcer treatment, and vigilance toward the risk of hypoglycemia in this population is thus necessary.

## Background

The incidence of type 2 diabetes mellitus (DM) has risen globally, from 108 million cases in 1980 to 422 million cases in 2014. In 2019, diabetes was the ninth leading cause of death in the world, directly causing an estimated 1.5 million deaths. Unmanaged glycemic control, a major threat to patients with DM, causes macrovascular and microvascular complications. However, adverse reactions to some antihyperglycemic medications that can cause hypoglycemia might cause fatal events such as comas, seizures, life-threatening arrhythmias, myocardial infarctions, and death [1]. Several antibiotics have been documented to cause hypoglycemic episodes [2–4]. Furthermore, giving antibiotics to patients taking sulfonylureas might increase the risk of hypoglycemia [5–7]. Therefore, clinicians must know whether antibiotics might increase the risk of severe hypoglycemic events among patients with diabetes.

The prevalence of *Helicobacter pylori* infection also remains high in most countries, in which more than half of the global population lives [8]. *H. pylori* infection increases the incidence of diabetes due to its increased levels of C-reactive protein and interleukin 6 (IL-6), which results in insulin resistance [9]. Several studies on people without diabetes have demonstrated positive associations between *H. pylori* infection, metabolic syndromes, and hyperglycemia [10]. Therefore, eradicating *H.pylori* infections in patients with DM is vital. The standard treatment for *H. pylori* infection is triple therapy, which involves amoxicillin, clarithromycin, and a proton pump inhibitor (PPI). However, several case reports have revealed hypoglycemia can occur when these regiments are in patients taking diabetes medication [11]. Another case report described an 82-year-old man with type 2 diabetes using insulin detemir who experienced severe hypoglycemia during treatment for *H. pylori* infection [12].

Therefore, we hypothesized that patients with diabetes might experience a greater risk of hypoglycemic episodes when treated with the triple therapy for the eradication of *H. pylori*. This study analyzed the incidence of severe hypoglycemia in patients with diabetes while being treated for *H. pylori* infection in a national database.

## Method

### Institutional review board approval

This study was approved by the Joint Institutional Review Board of Taipei Medical University (TMU-JIRB No. 202323079), and a waiver of informed consent was granted due to the study design.

### Data source and study design

The Taiwan National Health Insurance Research Database (NHIRD), managed by the Health and Welfare Data Science Center (HWDC), Ministry of Health and Welfare, Taiwan, was used in this retrospective cohort study. The NHIRD is a collection of claims-based data that records the reimbursement information of beneficiaries covered by the Taiwan National Health Insurance (NHI) legislation, which includes 99% of Taiwan’s residents. The database contains data on inpatient, outpatient, and pharmaceutical claims, as well as disease diagnoses coded using the International Classification of Diseases, Ninth Revision, Clinical Modification (ICD-9-CM) and the International Classification of Diseases, Tenth Revision (ICD-10) since 2016. The database also includes beneficiary and provider enrollment information. The NHIRD is linked to the National Death Registry to retrieve death records, and this linkage is governed by HWDC regulations.

### Participants

Patients having a diagnosis of DM with at least two diagnostic claims (ICD-9-CM: 255.0) and prescription claims for antidiabetic medications at any time between 2002 and 2016 were selected for this study. Patients were excluded from the study if information on their sex was missing from the NHIRD, if they were aged less than 18 years, or if they had a history of malignant tumors during the study period. Additionally, we excluded patients diagnosed with type 1 DM, end-stage renal disease (ESRD), or chronic liver disease. Among the patients selected for this study, we enrolled patients with type 2 DM and with at least one diagnostic claim of peptic ulcer disease (ICD-9: 531–533) at any time from 2002 to 2016. Finally, patients who experienced hypoglycemic episodes, including hypoglycemic shock (ICD-9: 250.8), hypoglycemic coma (ICD-9: 251.0), hyperinsulinism hypoglycemia (ICD-9:251.1), and unspecified hypoglycemia (ICD-9: 251.2), during the study were matched at a 1:2 ratio with patients who did not experience hypoglycemic episodes.

### Study outcome

The study outcome was the adjusted odds ratio (aOR) of exposure to triple therapy—PPI (Anatomical Therapeutic Chemical code, ATC code: A02BC), amoxicillin (ATC code: J01CA04), and clarithromycin (ATC code: J01FA09)—1 year before a hypoglycemic episode. To further authenticate whether the prescription of each of these three medications was used as triple therapy treatment of *H. pylori* eradication, we separated the study participants into four groups based on the interval between the exposure of these medications and the hypoglycemic episode as follows: within 30 days, 30 to 90 days, 90 to 180 days, and more than 180 days.

### Statistical analysis

Baseline characteristics were analyzed using the standardized mean difference (SMD). An SMD of ≥ 0.1 indicated a non-negligible difference between the two groups. All analyses were performed using SAS/STAT version 9.4 (SAS Institute Inc., Cary, NC, USA) and STATA 14 (Stata Corp LP, College Station, TX, USA). A *p* value of < 0.05 was considered statistically significant.

## Results

**Fig. 1:**
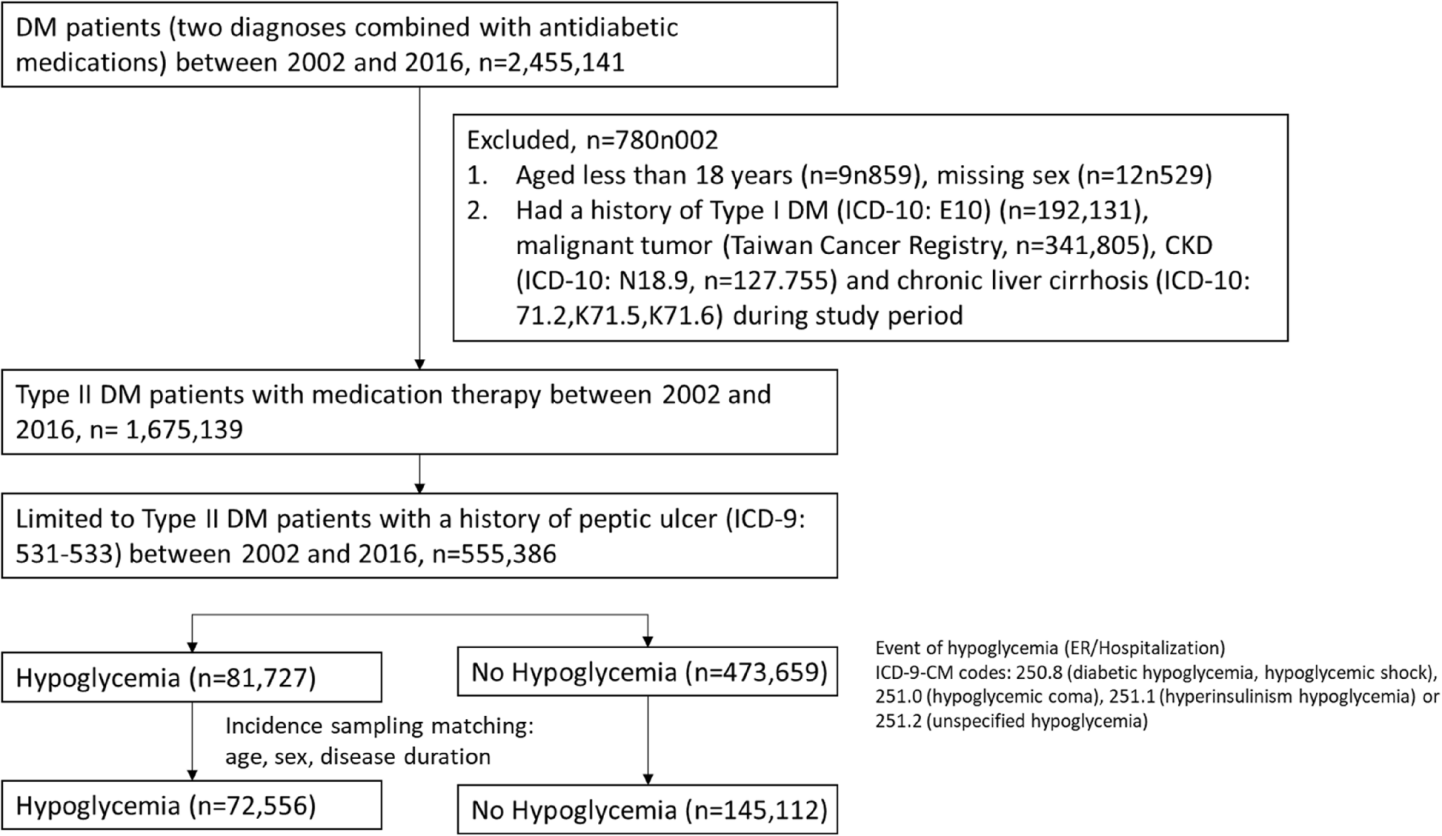
The selection process of the study participants. DM, diabetes mellitus; CKD, chronic renal disease; ICD, International classification of diseases

As illustrated in Fig. 1, a total number of 2,455,141 patients who had two diagnoses of DM and were prescribed antidiabetic medication from 2002 to 2016 were selected from our database. In total, 780,002 patients were excluded because they had missing information on their sex; were younger than 18 years old; had type 1 DM; or had a history of malignant tumors, ESRD, or chronic liver disease. Of the remaining 1,675,139 patients with DM, 555,386 patients had a history of peptic ulcers during that period. Among this group of patients with DM, we identified 81,727 patients who had hypoglycemic episodes and 473,659 patients who did not experience hypoglycemic episodes. The 1:2 propensity score matching was based on age, sex, and the duration. Finally, 72,556 patients who had hypoglycemic episodes and 145,112 patients who did not were selected. Among these selected patients, there was a significant difference in the number of comorbidities between patients with/without hypoglycemia (Table 1). Considering some medications, such as insulin, sulfonylurea, and anti-depressants are known to be associated with hypoglycemia, we investigated the prescription record of selected patients, which found a significant lower exposure of all these medication in the hypoglycemic group.

**Table 1:** The demographic information of study participants

	Control	%	Case	%	SMD
Case number	145,112		72,556		
Mean age	65.23 ± 10.62		65.23 ± 10.62		0.001
Sex					
Male	62,454	43.0	31,227	43.0	0.000
Female	82,658	57.0	41,329	57.0	0.000
Comorbidity					
0	43,414	29.9	12,498	17.2	0.302
1 ~ 2	69,890	48.2	37,038	51.0	0.058
> 3	31,808	21.9	23,020	31.7	0.223
Medication					
Insulin	49,189	33.9	22,804	31.4	0.053
Sulfonylurea	95,719	66.0	40,011	55.1	0.223
Anti-depressant	32,861	22.6	8,841	12.2	0.278

*SMD* standardized mean difference

In the hypoglycemic group, PPI exposure within one year before the hypoglycemic episode was significantly higher than in the non-hypoglycemic group (aOR: 1.35, 95% confidence interval (CI): 1.31 to 1.38, *p* < 0.001) (Table 2). In addition, a clear association was found between the interval of PPI exposure and the hypoglycemic episode. Namely, the closer the interval of PPI exposure with the hypoglycemia, the higher the aOR of the event. Furthermore, the exposure of triple therapy—PPI, amoxicillin, and clarithromycin—led to a significantly higher aOR was in patients with diabetes and peptic ulcers who had hypoglycemic episodes than in patients who did not (aOR: 1.75, 95% CI 1.64 to 1.88, *p* < 0.001). Similarly, the highest aOR, 5.77 with a 95% CI of 4.82 to 6.92, was found in patients with diabetes and peptic ulcers within 30 days after triple therapy treatment.

**Table 2:** The adjust odds ratio (aOR) of hypoglycemia in diabetic patients with the exposure history of proton pump inhibitor (PPT) only, and the triple therapy (PPI + amoxicillin + clarithromycin)

	Control	%	Case	%	aOR	*p*-value
Sample size	145,112	100	72,556	100	217,668	
**PPI only**						
*Any use*						
Non-user	118,257	81.5	53,272	73.4	1.00 (Ref.)	
User	26,855	18.5	19.284	26.6	1.36 (1.31–1.38)	< 0.001
*By recency*						
Non-user	118,257	81.5	53,272	73.4	1.00(Ref.)	< 0.001
< 30 days	8,003	5.5	7,227	10.0	1.67(1.61–1.74)	< 0.001
31 ~ 90 days	58.862	4.0	4,425	6.1	1.42(1.36–1.48)	< 0.001
91 ~ 180 days	5,051	3.5	3,159	4.4	1.18(1.12–1.23)	< 0.001
> 180 days	7,939	5.5	4,473	6.2	1.07(1.03–1.11)	0.002
**Triple therapy**						
*Any use*						
Non-user	143,255	98.7	70,604	97.3	1.00(Ref.)	
User	1,857	1,3	1,952	2.7	1.75(1.64–1.88)	< 0.001
*By recency*						
Non-user	143,255	98.7	70,604	97.3	1.00(Ref.)	
< 30 days	163	0.1	562	0.8	7.00(5.87–8.33)	< 0.001
31 ~ 90 days	330	0.2	300	0.4	1.85(1.58–2.16)	< 0.001
91 ~ 180 days	477	0.3	346	0.5	1.48(1.29–1.70)	< 0.001
> 180 days	887	0.6	744	1.0	1.71(1.55–1.89)	< 0.001

## Discussion

Hypoglycemia is a serious concern for patients with DM. Refractory hypoglycemia if untreated can cause brain damage and other irreversible complications. The present study was initiated based on the clinical observation that hypoglycemic episodes can occur in patients with diabetes during treatment for *H. pylori*. The standard regimen for *H. pylori* eradication is triple therapy—with clarithromycin, amoxicillin, and a PPI. Any one of the three medications rarely causes hypoglycemia when used alone. However, in our study, hypoglycemia and triple therapy were significantly associated in our analysis of medical records in the NHIRD. Considering the large number of patients with diabetes who require *H. pylori* eradication for peptic ulcer treatment, physicians and patients should be aware of this increased risk and take measures to prevent hypoglycemia.

The antibiotic treatment used in *H. pylori* eradication affects the gut microbiota, which causes metabolic amelioration after the treatment is complete [13]. Clarithromycin is a macrolide antibiotic that is absorbed in the gastrointestinal tract. Several case reports have reported the occurrence of hypoglycemia when clarithromycin was combined with sulfonylurea [5]. A case report also reported severe hypoglycemia due to clarithromycin-repaglinide drug interaction [14]. It was suggested that clarithromycin increased the bioavailability of repaglinide due to CYP3A4 inhibition and p-glycoprotein inhibition. In addition, a study proposed that the absorption of clarithromycin is greater when omeprazole is used due to the increase in gastric pH from omeprazole [15].

Amoxicillin is a semisynthetic penicillin antibiotic; its most common adverse effect is gastrointestinal upset [16]. In addition, amoxicillin exerts extremely negative effects on the gut microbiome. A total elimination of aerobic gram-positive cocci and an associated increased resistance of enterobacteria has been observed when individuals are administered amoxicillin.

One study reported that omeprazole increased the duration of hypoglycemia and peak hypoglycemia induced by sulfonylureas in healthy albino rabbits [17]. PPIs can significantly raise serum gastrin concentration and affect glucose metabolism by promoting B-cell regeneration and expansion; PPIs can therefore increase the risk of hypoglycemia. Another study showed that omeprazole might increase the risk of hypoglycemia inpatients treated with gliclazide [18].

For those patients with diabetes with reasonable glycemic control, practitioners and patients should be explicitly warned of the risk of hypoglycemia during treatment for *H. pylori*.

One of the strengths of this study is the large number of study participants enrolled from the NHIRD, which has the medical records of almost all of the residents in Taiwan. Second, we enrolled patients with DM with a peptic ulcer into the control group to control for confounding from poor nutrition due to peptic ulcer. Third, we only enrolled patients with hypoglycemic episodes that were critical enough to require hospitalization. Therefore, patients with diabetes who have undergone *H. pylori* eradication should be vigilant against hypoglycemia.

On the other hand, the present study has several limitations. First, due to the availability of the data from NHIRD, the exposure of PPI, amoxicillin, and clarithromycin was not limited to the same prescription. However, because amoxicillin and clarithromycin is infrequently prescribed in adults, these three medications, especially within 1 month, are very likely to be used for *H. pylori*. Second, the data on glycemic control and other metabolic parameters, such as body mass index, glycated hemoglobin, and lipid profiles, were unavailable for this study. Lastly, the hypoglycemic groups exhibited significantly different baseline demographic information compared with age, sex and disease duration-matched controls. In order to prevent bias, aOR was utilized in the statistical analysis rather than the crude OR. In addition, in the hypoglycemic group, the exposure of insulin, sulfonylurea and anti-depressants were significantly lower, which are known to increase the risk of hypoglycemia in DM patients.

In conclusion, hypoglycemia is more common among patients with type 2 diabetes who receive *H. pylori* treatment, especially for those who are taking antihyperglycemic medications. Therefore, caution against severe hypoglycemia is warranted when patients with type 2 diabetes are undergoing treatment for *H. pylori*.

## Data Availability

Please contact the corresponding authors (CT Hong and LN Chien). Data and materials can be available upon permission from the TMU-JIRB.
